# Genetic Yield and Imaging Characteristics in Apical-Dominant Hypertrophic Cardiomyopathy

**DOI:** 10.1016/j.jacasi.2026.08.004

**Published:** 2026-10-06

**Authors:** Inki Moon, Mi-Ae Jang, Saerom Kim, Dong-Eon Kim, Hee-Dong Kim, Min Gyu Kong, Hyun Woo Park, Hyung Oh Choi, Jon Suh, Yoon Haeng Cho, Nae-Hee Lee, Hye-Sun Seo

**Affiliations:** a Division of Cardiology, Department of Internal Medicine, Soonchunhyang University Bucheon Hospital, Soonchunhyang University College of Medicine, Bucheon, Republic of Korea; b Department of Laboratory Medicine and Genetics, Samsung Medical Center, Sungkyunkwan University School of Medicine, Seoul, Republic of Korea; c Division of Cardiology, Department of Internal Medicine, Soonchunhyang University Cheonan Hospital, Soonchunhyang University College of Medicine, Cheonan, Republic of Korea; d Division of Cardiology, Department of Internal Medicine, Ewha Womans University Mokdong Hospital, Ewha Womans University College of Medicine, Seoul, Republic of Korea

**Keywords:** cardiac magnetic resonance, genotype-phenotype correlation, hypertrophic cardiomyopathy, whole-exome sequencing

Hypertrophic cardiomyopathy (HCM) is the most common inherited cardiac disease, characterized by unexplained left ventricular (LV) hypertrophy, with pathogenic (P) or likely pathogenic (LP) variants in sarcomeric genes identified in 30% to 60% of patients.^1^ Apical HCM, a distinct morphological subtype predominantly affecting the LV apex, is significantly more prevalent in Asian populations (up to 40% of cases) compared with Western cohorts (approximately 8%).^2^^,^^3^ However, genetic testing yield in apical HCM has historically been lower than in classic forms, with prior studies reporting yields of only 14% to 25% using targeted gene panels.^4^, ^5^, ^6^ Whole-exome sequencing (WES) may improve diagnostic sensitivity by enabling comprehensive analysis of all protein-coding regions.^7^ We aimed to evaluate the diagnostic yield of WES and genotype-phenotype associations, including cardiac magnetic resonance (CMR)–defined myocardial fibrosis, in a Korean HCM cohort with a high prevalence of apical morphology. Understanding the genetic landscape of apical HCM is important for informing family screening strategies and risk stratification in Asian populations.

We prospectively enrolled 116 unrelated, consecutive HCM patients (LV wall thickness ≥15 mm by echocardiography) from Soonchunhyang University Bucheon Hospital between December 2021 and May 2024. Patients with uncontrolled hypertension, severe valvular disease, or concomitant systemic disease were excluded. All patients underwent WES; genomic DNA was extracted from peripheral blood and sequenced on an Illumina platform. Variants were classified according to American College of Medical Genetics and Genomics/Association for Molecular Pathology guidelines^8^; patients harboring P/LP variants were classified as genotype-positive. Apical HCM was defined as hypertrophy predominantly involving the LV apex, including both pure apical and mixed (apical with midventricular involvement) types.^3^ A subset of patients (n = 57) underwent CMR. Late gadolinium enhancement (LGE) extent and extracellular volume (ECV) fraction were quantified using validated automated deep-learning software (Myomics, Phantomics Inc).^9^ Continuous variables were compared using the Student's *t*-test or Wilcoxon rank-sum test, and categorical variables were compared using the chi-square or Fisher exact test. This study was approved by the Soonchunhyang University Bucheon Hospital Institutional Review Board (SCHBC 2021-10-003-001), and all participants provided written informed consent.

The mean age at diagnosis was 57.6 ± 12.9 years, and 30.2% (35/116) were female. Most patients (103/116; 88.8%) were in NYHA functional class I. Apical morphology was present in 82 of 116 patients (70.7%). Overall, P/LP variants were identified in 42 of 116 patients (36.2%) across 10 genes. Across these 42 genotype-positive patients, we identified 28 distinct P/LP variants spanning 10 genes (Table 1). *MYBPC3* was the most frequently mutated gene (17/42; 40.5% of genotype-positive patients), followed by *MYH7* (10/42; 23.8%); together, these 2 genes accounted for 64.3% (27/42) of genotype-positive cases (Table 1). Sarcomeric gene variants accounted for 92.9% (39/42) of positive cases, with thick-filament genes (*MYBPC3*, *MYH7*, *MYL3*, *MYH6*) representing 69.0% (29/42). When comparing gene spectra between apical and nonapical genotype-positive patients, no significant differences were observed (*P* = 0.32, Fisher exact test; Table 1). Nonsarcomeric P/LP variants were identified in *CACNA1C* and *PRKAG2* (1 patient each). Among patients with apical morphology, P/LP variants were identified in 26 of 82 (31.7%), and specifically in 11 of 49 (22.4%) with pure apical type. Pure apical morphology was significantly more frequent in genotype-negative patients (51.4% [38/74] vs 26.2% [11/42]; *P* = 0.015). Genotype-positive patients were younger at diagnosis (53.2 ± 13.9 vs 59.7 ± 12.5 years; *P* = 0.015), and had greater maximal wall thickness (18.9 ± 3.4 vs 17.4 ± 2.7 mm; *P* = 0.008), thicker interventricular septum (14.8 ± 4.3 vs 12.8 ± 3.2 mm; *P* = 0.017), and lower LV ejection fraction (61.8% ± 7.6% vs 65.8% ± 5.7%; *P* = 0.008). Other clinical characteristics, including hypertension, diabetes mellitus, and atrial fibrillation, did not differ significantly between groups.

**Table 1:** Pathogenic and Likely Pathogenic Variants Identified by Whole-Exome Sequencing in 42 Genotype-Positive Hypertrophic Cardiomyopathy Patients

Gene/Variant	Class	Genotype-positive (N = 42)	Apical (n = 26)	Nonapical (n = 16)	ACMG Criteria
Genes encoding thick myofilament of sarcomere					
MYBPC3 (NM_000256.3)		17 (40.5)	10 (38.5)	7 (43.8)	
c.2833_2834del/p.Arg945Glyfs∗105	P	3	2	1	PS4_M, PVS1, PM2
c.2273 G > A/p.Gly758Asp	LP	3	2	1	PS4_M, PM2, PM5, PP2, PP3
c.86del/p.Phe29Serfs∗10	P	2	1	1	PS4_P, PVS1, PM2
c.3794_3797del/p.Glu1265Alafs∗65	LP	1	0	1	PVS1_S, PM2
c.3677dup/p.Phe1227Leufs∗15	LP	1	1	0	PVS1, PM2
c.3624del/p.Lys1209Serfs∗28	P	1	1	0	PS4_M, PVS1, PM2
c.3065 G > A/p.Arg1022His	LP	1	1	0	PM2, PM5, PS4_P, PP2
c.3015_3036dup/p.Pro1013Aspfs∗45	LP	1	0	1	PVS1, PM2
c.2459 G > A/p.Arg820Gln	LP	1	1	0	PS4_M, PM2, PM5, PS3_P, PP2
c.2164 G > T/p.Glu722∗	LP	1	0	1	PVS1, PM2
c.1483 C > T/p.Arg495Trp	P	1	1	0	PS4, PM1, PM2, PM5, PP2, PP3
c.1113_1116del/p.Ala372Thrfs∗3	LP	1	0	1	PVS1, PM2
MYH7 (NM_000257.4)		10 (23.8)	6 (23.1)	4 (25.0)	
c.2608 C > T/p.Arg870Cys	P	3	3	0	PS4, PM1, PM2, PM5, PP3
c.1350 G > T/p.Lys450Asn	LP	2	1	1	PS4_M, PM1, PM2, PM5, PP3
c.4130 C > T/p.Thr1377Met	P	1	0	1	PS4, PM2, PP1_S, PP3
c.2680 G > A/p.Glu894Lys	LP	1	0	1	PS4_M, PM1, PM2, PM5, PP3
c.1988G > A/p.Arg663His	P	1	1	0	PP1_S, PS4, PM1, PM2
c.1816 G > T/p.Val606Leu	LP	1	1	0	PM1, PM2, PM5, PP3
c.1324 C > T/p.Arg442Cys	P	1	0	1	PP1_S, PM1, PM2, PP3
MYL3 (NM_000258.3)		1 (2.4)	1 (3.8)	0 (0.0)	
c.281 G > A/p.Arg94His	LP	1	1	0	PS4_M, PM2, PP1, PP2
MYH6 (NM_002471.3)		1 (2.4)	1 (3.8)	0 (0.0)	
c.3568_3572del/p.Thr1190Argfs∗41	LP	1	1	0	PVS1, PM2
Genes encoding thin myofilament of sarcomere					
TNNI3 (NM_000363.5)		5 (11.9)	5 (19.2)	0 (0.0)	
c.434 G > A/p.Arg145Gln	P	4	4	0	PS3, PM2, PM5, PP2, PP3
c.422 G > A/p.Arg141Gln	P	1	1	0	PS2, PM2, PM5, PP1, PP2
TPM1 (NM_001365778.1)		4 (9.5)	2 (7.7)	2 (12.5)	
c.64 G > A/p.Ala22Thr	LP	4	2	2	PS4_M, PM2, PP2, PP3
TNNT2 (NM_001276345.2)		1 (2.4)	0 (0.0)	1 (6.3)	
c.304 C > T/p.Arg102Trp	P	1	0	1	PS3, PM2, PM5, PP2, PP3
Genes encoding sarcomere-associated M-band protein					
ALPK3 (NM_020778.5)		1 (2.4)	1 (3.8)	0 (0.0)	
c.691 C > T/p.Gln231∗	LP	1	1	0	PVS1, PM2
Genes associated with syndromic left ventricular hypertrophy					
CACNA1C (NM_000719.7)		1 (2.4)	0 (0.0)	1 (6.3)	
c.1552 C > T/p.Arg518Cys	LP	1	0	1	PM2, PM5, PP1, PP3
PRKAG2 (NM_016203.4)		1 (2.4)	0 (0.0)	1 (6.3)	
c.905 G > A/p.Arg302Gln	P	1	0	1	PS3_M, PS2, PM2, PP1, PP3
Total		42 (100.0)	26 (61.9)	16 (38.1)	

Values are presented as n (%) for gene rows and as n for variant rows. All carriers were heterozygous. ∗ is the standard HGVS nomenclature indicating a stop codon.

ACMG = American College of Medical Genetics and Genomics; LP = likely pathogenic; P = pathogenic.

In the CMR subgroup (n = 57; genotype-positive: 18, genotype-negative: 39), genotype-positive patients had significantly greater LGE extent (median: 7.5% [IQR: 3.5-12.3] vs 2.4% [IQR: 1.0-3.7]; *P* = 0.002), more LGE-involved segments (median: 7 [IQR: 5-11] vs 4 [IQR: 3-6]; *P* = 0.002), and higher ECV fraction (median: 30.6% [IQR: 27.2%-34.1%] vs 27.2% [IQR: 25.2%-28.7%]; *P* = 0.015), despite no significant difference in CMR-measured maximal wall thickness (21.3 ± 5.0 vs 18.6 ± 4.4 mm; *P* = 0.060).

In this Korean HCM cohort with a high prevalence of apical morphology (82/116; 70.7%), WES identified P/LP variants in approximately 36% (42/116) of patients, with a notable yield of 31.7% (26/82) even in apical HCM. These findings represent a substantial improvement over prior reports using targeted gene panels (14%-25%),^4^, ^5^, ^6^ supporting the clinical value of comprehensive genomic analysis regardless of morphological subtype. The similar distribution of causative genes between apical and nonapical groups suggests shared genetic architecture across HCM morphologies. Notably, pure apical morphology was significantly less common in genotype-positive than genotype-negative patients (26.2% [11/42] vs 51.4% [38/74]; *P* = 0.015), suggesting this distinctive subtype may involve mechanisms beyond classic sarcomeric variants, such as copy number variations or noncoding contributors not captured by standard WES. The high proportion of apical HCM in our cohort likely reflects the known epidemiological pattern in East Asian populations, though referral bias at a tertiary center may have contributed.

Genotype-positive patients demonstrated earlier disease onset, greater hypertrophy, and a significantly higher burden of myocardial fibrosis, consistent with observations from large international registries.^10^ The association between genotype status and CMR-defined fibrosis, including both LGE extent and ECV fraction, may have important implications for arrhythmic and heart failure risk stratification in HCM.^5^ Given that myocardial fibrosis is a recognized predictor of adverse outcomes in HCM, the greater fibrosis burden observed in genotype-positive patients underscores the value of integrating genetic information with advanced imaging for individualized risk assessment. Identifying a P/LP variant in the proband—even with apical morphology—allows systematic evaluation of family members who may harbor the same variant and may benefit from early surveillance and intervention.

Regarding study limitations, this was a single-center study of exclusively Korean patients from a tertiary referral center, which may explain the high proportion of apical HCM and may limit generalizability. The modest sample size, particularly in the CMR subgroup (n = 57), limits statistical power for subgroup analyses. As only 49% (57/116) of patients underwent CMR, selection bias toward more severe phenotypes cannot be excluded. The cross-sectional design precludes assessment of prognostic outcomes. The nonapical comparator group comprised only 34 patients, which limits the precision of intermorphology gene-distribution comparisons and renders these comparisons exploratory. Additionally, our WES analysis focused on coding regions and did not assess copy number variations or deep intronic variants, which may contribute to genotype-negative cases.

## Funding Support and Author Disclosures

This work was supported by a National Research Foundation of Korea grant funded by the Korea government (NRF-2021R1G1A1093522) and the Soonchunhyang University Research Fund. The authors have reported that they have no relationships relevant to the contents of this paper to disclose.
